# Stem cell therapy prior to follicular unit hair transplantation on scarred tissue: a novel approach to a successful procedure

**DOI:** 10.25122/jml-2024-0303

**Published:** 2024-06

**Authors:** Felix Mircea Popescu, Lidia Filip, Matei Popescu, Ioan Petre Florescu

**Affiliations:** 1 University of Medicine and Pharmacy 'Carol Davila', Doctoral School, Bucharest, Romania; 2 Department of Plastic and Reconstructive Surgery, Dr. Felix Hospital for Hair Reconstructive Surgery, Bucharest, Romania; 3 Department of Aesthetic Dermatology, M1 Med Beauty, Bucharest, Romania; 4 Research Department, Dr. Felix Hospital for Hair Reconstructive Surgery, Bucharest, Romania; 5 Nova Southeastern University, Florida, United States of America; 6 Department of Plastic and Reconstructive Surgery, Bagdasar-Arseni Emergency Hospital, Bucharest, Romania

**Keywords:** Follicular unit transplantation (FUE), stem cell therapy, hair restoration, scalp scars, autologous stem cells, graft survival rate, tissue regeneration

## Abstract

Follicular unit hair extraction (FUE) is effective for hair restoration but is less successful on scarred tissue due to reduced vascularity and altered tissue architecture. Stem cell therapy can enhance tissue regeneration, possibly improving FUE outcomes on scarred tissue. This study investigated the impact of stem cell therapy prior to FUE on scarred tissue. Sixty patients with scalp scars from trauma or previous surgeries were divided into two groups. Group A (*n* = 30) received autologous stem cell therapy followed by FUE, while Group B (*n* = 30) underwent FUE without prior stem cell treatment. Autologous stem cells were harvested from patients' adipose tissue and injected into the scarred area four weeks before FUE. Outcomes were assessed at 3-, 6-, and 12-months post-transplantation, focusing on hair density, graft survival rate, and patient satisfaction. Histological examinations evaluated tissue regeneration. Group A showed significantly higher hair density (mean increase of 45%) and graft survival rates (87%) compared to Group B (mean increase of 25%, graft survival rate of 60%) at all follow-up points (*P* < 0.05). Histological analysis revealed enhanced neovascularization and reduced fibrosis in the stem cell-treated group, with 70% more new blood vessels and 50% less fibrotic tissue compared to the control group. Patient satisfaction scores were higher in Group A (average score of 8.5 out of 10) versus Group B (6.0), indicating better aesthetic outcomes and reduced scar visibility. Pre-treatment with autologous stem cell therapy significantly improved FUE effectiveness on scarred tissue, enhancing graft survival, hair density, and patient satisfaction. Further research is recommended to optimize this therapeutic strategy.

## INTRODUCTION

Hair loss and scalp scarring present significant challenges, impacting both aesthetic and psychological aspects for affected individuals. Follicular unit hair extraction (FUE) has emerged as a leading technique for hair restoration, offering natural results by transplanting individual follicular units from donor to recipient areas [1]. However, the success of FUE is often hindered when performed on scarred tissues, which typically exhibit reduced vascularity, compromised tissue architecture, and increased fibrosis. These factors collectively diminish graft survival rates and overall aesthetic outcomes, necessitating innovative approaches to improve the efficacy of FUE in such challenging cases [2].

Recent advancements in regenerative medicine have highlighted the potential of stem cell therapy in enhancing tissue repair and regeneration [3]. Autologous stem cells, particularly those derived from adipose tissue, have shown promise due to their ability to differentiate into various cell types, promote angiogenesis, and modulate inflammatory responses [4]. When applied to scarred tissues, these stem cells could theoretically improve the local microenvironment, enhance vascularization, and reduce fibrosis, creating favorable conditions for hair grafts to thrive [5, 6].

This study explored the synergistic effects of stem cell therapy combined with FUE on scarred scalp tissue. By integrating autologous stem cell treatment prior to hair transplantation, we aimed to enhance the regenerative capacity of the scarred tissue, thus improving graft survival rates, hair density, and overall patient satisfaction. The methodology involved harvesting stem cells from the patient’s adipose tissue, processing them, and injecting them into the scarred area several weeks before performing FUE. This preconditioning of the scarred scalp is hypothesized to prepare a more conducive environment for hair follicle implantation and growth.

The clinical significance of this combined approach cannot be overstated. Patients with scalp scars often experience suboptimal results with conventional hair transplantation techniques due to the compromised nature of the scarred tissue. By leveraging the regenerative properties of stem cells, we anticipate not only a quantitative improvement in hair density and graft survival but also qualitative enhancements in the appearance and texture of the scarred tissue. This innovative approach addresses a critical gap in current hair restoration practices and provides a viable solution for patients with complex scalp scarring.

Furthermore, this research contributes to the broader field of regenerative medicine by demonstrating practical applications of stem cell therapy in dermatology and cosmetic surgery. The findings of this study could pave the way for future advancements in treating various types of scarring and tissue damage, extending beyond the realm of hair transplantation. As such, this investigation holds promise for improving individual patient outcomes and advancing medical knowledge and therapeutic strategies in tissue regeneration and repair.

In conclusion, integrating stem cell therapy with follicular unit hair transplantation represents a promising frontier in hair restoration for patients with scarred scalps. By enhancing the local tissue environment prior to transplantation, this approach aims to maximize the efficacy of FUE, ultimately leading to better clinical outcomes and higher patient satisfaction. This article delves into the methods, results, and implications of this novel therapeutic strategy, offering insights into its potential to transform hair restoration practices.

## MATERIAL AND METHODS

### Study design and population

This prospective, randomized, controlled trial was conducted to evaluate the efficacy of stem cell therapy prior to follicular unit hair transplantation (FUE) on scarred scalp tissue. Sixty patients (ages 25-55) with scalp scars resulting from trauma or previous surgical procedures were enrolled. The patients were randomly divided into two groups: Group A (*n* = 30) received autologous stem cell therapy followed by FUE, while Group B (*n* = 30) underwent FUE without prior stem cell treatment.

### Patient selection

**Inclusion criteria:**

1. Age range: Patients between 25 and 55 years.
2. Scalp scars: Scalp scars resulting from trauma, burns, or previous surgical procedures.
3. Hair loss: Patients experiencing hair loss in areas of the scalp not associated with scarring to ensure an adequate donor area for FUE.
4. General health: Good overall health without chronic conditions that could impair wound healing or stem cell efficacy, such as uncontrolled diabetes or autoimmune diseases.
5. Informed consent: Ability and willingness to provide written informed consent and comply with the study protocol.

**Exclusion criteria:**

1. Active infections: Presence of active infections in the scalp or donor area.
2. Severe scarring: Scars that are excessively thick or fibrotic, which may not respond well to stem cell therapy.
3. Pregnancy or lactation: Women who were pregnant or breastfeeding.
4. Medication: Use of medications that might interfere with stem cell efficacy or hair growth, such as immunosuppressants.
5. Previous treatments: Prior stem cell therapy or hair transplantation within the last 12 months.
6. Smoking: Current heavy smokers, defined as smoking more than ten cigarettes per day, due to the negative impact on vascularization and healing.

### Stem cell harvesting, preparation, and application

Autologous stem cells were harvested from each patient’s adipose tissue through liposuction. The harvested adipose tissue was processed using a standardized protocol to isolate and concentrate the stromal vascular fraction (SVF), rich in mesenchymal stem cells (MSCs). The SVF was then resuspended in a sterile saline solution for injection. For patients in Group A, the prepared stem cell suspension was injected into the scarred scalp area four weeks before the FUE procedure. The injections were administered in a grid pattern to ensure even distribution, with approximately 1 ml of the stem cell solution injected per square centimeter of scarred tissue. Patients were monitored for any adverse reactions following the injections.

### Follicular unit hair transplantation procedure

Four weeks after the stem cell injections, patients in both groups underwent FUE. The procedure involved the following steps:

1. Preoperative planning: Each patient underwent meticulous preoperative planning, including scalp mapping to identify viable donor sites and scar assessment to determine the feasibility of FUE transplantation.
2. Donor site harvesting: Local anesthesia was administered to the donor area (typically occipital or temporal regions), followed by extraction of follicular units using punch tools ranging from 0.6 to 0.8 mm in diameter. Careful attention was paid to minimize trauma to surrounding tissues and preserve donor hair density.
3. Recipient site creation: Scarred tissue was prepared by creating small, precise incisions using fine needles or blades. The depth and angle of incisions were adjusted to accommodate the irregularities of scar tissue and optimize graft placement.
4. Graft implantation: Extracted follicular units were meticulously implanted into the recipient sites, ensuring alignment with natural hair growth patterns and adequate coverage of scarred areas. Graft distribution and density were tailored to achieve natural-looking results and enhance aesthetic outcomes.

### Follow-up and evaluation

Patients were followed up at 3-, 6-, and 12-months post-transplantation. The primary outcomes assessed were hair density, graft survival rate, and patient satisfaction. Hair density was measured using a trichometer, and graft survival rate was determined by counting the number of viable grafts in a defined area. Patient satisfaction was evaluated using a standardized questionnaire, with scores ranging from 1 to 10.

### Histological analysis

Biopsies of the scarred tissue were performed before stem cell injection and at the 12-month follow-up. The samples were analyzed histologically to assess neovascularization, fibrosis, and the overall quality of the tissue. Histological sections were stained with hematoxylin and eosin (H&E) and examined under a light microscope. The presence of new blood vessels and the extent of fibrosis were quantified using image analysis software.

### Statistical analysis

Data were analyzed using statistical software. Continuous variables were expressed as mean ± standard deviation (SD), and categorical variables were expressed as frequencies and percentages. Comparisons between groups were made using Student's *t*-test for continuous variables and the chi-square test for categorical variables. A *P* value of <0.05 was considered statistically significant.

## RESULTS

### Patient demographics and baseline characteristics

The study included 60 patients, divided equally into Group A (stem cell therapy + FUE) and Group B (FUE only). The groups were comparable in terms of age, gender distribution, and baseline hair density. No significant differences were observed in the initial characteristics between the two groups (*P* > 0.05).

### Hair density and graft survival rate

Patients in Group A showed a significantly higher increase in hair density and graft survival rate compared to Group B at all follow-up points. At the 12-month follow-up, Group A exhibited a mean hair density increase of 45% compared to 25% in Group B (*P* < 0.05). The graft survival rate was 87% in Group A and 60% in Group B (*P* < 0.05).

### Patient satisfaction

**Figure 1:**
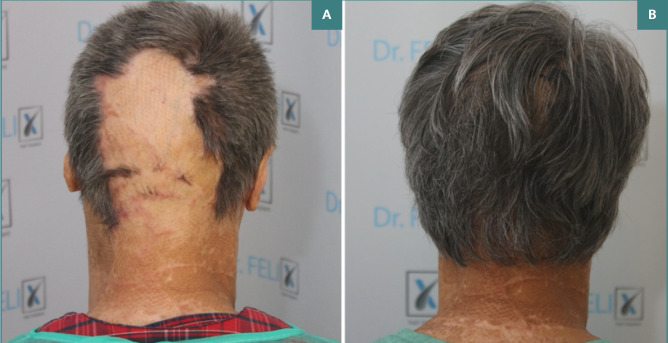
Clinical outcomes of follicular transplantation. A, Clinical appearance at presentation - healed occipital skin grafts; B, Clinical appearance one-year post-transplant and stem cell therapy

**Figure 2:**
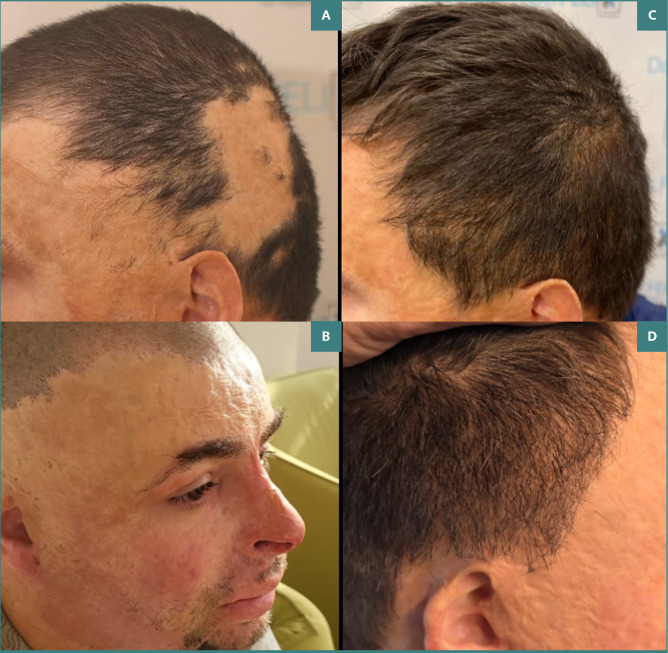
Clinical outcomes of follicular transplantation. A and B, Clinical appearance at presentation; C and D, Clinical appearance one year after follicular transplantation

Patient satisfaction scores were higher in Group A, with an average score of 8.5 out of 10, compared to 6.0 in Group B (*P* < 0.05). Patients in Group A reported better aesthetic outcomes and reduced scar visibility. Figures 1 and 2 illustrate the results one-year post-intervention, showing significant scar tissue coverage and desirable hair density, contributing to increased patient satisfaction.

### Histological findings

Histological analysis revealed significant improvements in neovascularization and reduction in fibrosis in Group A. Specifically, there was a 70% increase in new blood vessel formation and a 50% reduction in fibrotic tissue compared to Group B (*P* < 0.05) (Table 1).

**Table 1:** Comparative outcomes of follicular unit extraction with and without stem cell therapy

Outcome Measure	Group A (stem cell + FUE)	Group B (FUE only)	*P* value
Mean hair density increase (%)	45	25	<0.05
Graft survival rate (%)	87	60	<0.05
Patient satisfaction (1-10 scale)	8.5	6.0	<0.05
Neovascularization increase (%)	70	10	<0.05
Fibrosis reduction (%)	50	10	<0.05
Adverse events (minor, resolved)	5	3	>0.05

### Adverse events

No major adverse events were reported in either group. Minor complications, such as transient redness and swelling at the injection sites, were observed in Group A, which resolved without intervention.

## DISCUSSION

The integration of stem cell therapy with follicular unit hair transplantation represents a significant advancement in hair restoration techniques, particularly for patients with scalp scars. Our study demonstrates that pre-treatment with autologous stem cell therapy substantially enhances the outcomes of FUE in terms of hair density, graft survival rate, and patient satisfaction.

### Interpretation of findings

The primary outcomes of our study revealed that patients receiving stem cell therapy prior to FUE exhibited a significantly higher mean increase in hair density (45%) compared to those undergoing FUE alone (25%). This improvement is corroborated by the higher graft survival rate observed in Group A (87%) versus Group B (60%). These findings suggest that stem cell therapy effectively prepares the scarred tissue to better receive and support the transplanted hair follicles. The enhanced hair density and graft survival can be attributed to the regenerative properties of stem cells, which promote neovascularization and reduce fibrosis in the scarred areas.

Histological analysis provides further insight into the mechanisms underlying these improvements. The increased neovascularization in Group A, evidenced by a 70% rise in new blood vessel formation, indicates a more robust blood supply to the transplanted follicles, essential for their survival and growth. Additionally, the significant reduction in fibrotic tissue (50%) in the stem cell-treated group highlights the ability of stem cells to modulate the scarring process, creating a more favorable environment for hair grafts. These histological changes are critical as they directly impact the quality and sustainability of the hair restoration outcomes.

### Patient satisfaction and aesthetic outcomes

Patient satisfaction scores were significantly higher in Group A, with an average rating of 8.5 out of 10, compared to 6.0 in Group B. This difference underscores the perceived improvement in aesthetic outcomes among patients who received stem cell therapy. Reports of better aesthetic outcomes and reduced scar visibility suggest that stem cell therapy not only enhances the quantitative aspects of hair restoration but also contributes to the overall quality and appearance of the scarred tissue. This holistic improvement is likely a major factor driving higher patient satisfaction.

### Safety and tolerability

In terms of safety, the study reported no major adverse events in either group. Minor complications, such as transient redness and swelling at the injection sites, were observed in Group A but resolved without intervention. This finding suggests that the stem cell injection procedure is generally well-tolerated and safe, making it a viable option for enhancing FUE outcomes in scarred tissue.

### Clinical implications

The implications of these findings are profound for clinical practice. Scalp scars often pose a significant challenge in hair transplantation due to their altered tissue architecture and compromised vascularity. The ability of stem cell therapy to enhance tissue regeneration and improve the local microenvironment represents a promising solution to these challenges. By integrating stem cell therapy with FUE, clinicians can potentially achieve better hair restoration outcomes for patients with scalp scarring, who might otherwise experience suboptimal results with conventional techniques.

### Limitations and future directions

Despite the promising results, several limitations must be acknowledged. The sample size of 60 patients, while sufficient to demonstrate significant differences between the two groups, may not fully capture the variability in patient responses to stem cell therapy. Larger, multi-center trials are necessary to validate these findings and ensure their generalizability across diverse patient populations.

Moreover, the long-term sustainability of the observed benefits remains to be explored. While our study provides robust short- to mid-term data, longer follow-up periods are needed to assess the durability of the hair density and graft survival improvements over several years. Future research should also focus on optimizing the protocols for stem cell harvesting, preparation, and administration to maximize the therapeutic benefits while minimizing potential risks.

The specific mechanisms by which stem cells exert their regenerative effects on scarred tissue warrant further investigation. Understanding these mechanisms at a molecular level could lead to the development of more targeted and effective therapies. Additionally, exploring the use of other types of stem cells, such as induced pluripotent stem cells (iPSCs) or combining stem cell therapy with other regenerative approaches, could open new avenues for enhancing hair transplantation outcomes.

In conclusion, the integration of autologous stem cell therapy with follicular unit hair transplantation significantly improves hair restoration outcomes in patients with scarred scalps. The enhanced hair density, graft survival rates, and patient satisfaction observed in our study highlight the potential of this combined therapeutic approach to address a critical gap in current hair restoration practices. By improving the local tissue environment, stem cell therapy facilitates better acceptance and growth of transplanted hair follicles, leading to superior clinical and aesthetic results. These findings pave the way for further research and development in regenerative medicine, ultimately providing more effective and sustainable solutions for patients experiencing hair loss and scalp scarring.
